# The human side of hypoxia-inducible factor

**DOI:** 10.1111/j.1365-2141.2008.07029.x

**Published:** 2008-05

**Authors:** Thomas G Smith, Peter A Robbins, Peter J Ratcliffe

**Affiliations:** 1 Department of Physiology, Anatomy and Genetics, University of Oxford Oxford, UK; 2 Nuffield Department of Clinical Medicine, University of Oxford Oxford, UK

**Keywords:** erythropoietin, erythropoiesis, polycythaemia, iron, oxygen sensing

## Abstract

When humans are exposed to hypoxia, systemic and intracellular changes operate together to minimise hypoxic injury and restore adequate oxygenation. Emerging evidence indicates that the hypoxia-inducible factor (HIF) family of transcription factors plays a central regulatory role in these homeostatic changes at both the systemic and cellular levels. HIF was discovered through its action as the transcriptional activator of erythropoietin, and has subsequently been found to control intracellular hypoxic responses throughout the body. HIF is primarily regulated by specific prolyl hydroxylase-domain enzymes (PHDs) that initiate its degradation via the von Hippel-Lindau tumour suppressor protein (VHL). The oxygen and iron dependency of PHD activity accounts for regulation of the pathway by both cellular oxygen and iron status. Recent studies conducted in patients with rare genetic diseases have begun to uncover the wider importance of the PHD-VHL-HIF axis in systems-level human biology. These studies indicate that, in addition to regulating erythropoiesis, the system plays an important role in cardiopulmonary regulation. This article reviews our current understanding of the importance of HIF in human systems-level physiology, and is modelled around the classic physiological response to high-altitude hypoxia.

The human response to hypoxia is characterised by systemic changes in haematopoietic, respiratory, and cardiovascular physiology that combine to restore adequate oxygenation. Uptake and transport of oxygen are initially maximised through an increase in ventilation and cardiac output, while oxygen carriage is subsequently optimised through acceleration of erythropoiesis. The homeostatic drive underlying these systemic effects is similarly evident at the cellular level, where hypoxia is met by a coordinated transcriptional response that seeks to minimise hypoxic injury and redress the oxygen debt. This cellular response is controlled by the hypoxia-inducible factor (HIF) family of transcription factors, a DNA binding complex that was first defined as a regulator of erythropoietin (Epo) gene (*EPO*) expression but which is now known to be conserved in all animal species, irrespective of the presence of specialised oxygen transfer systems such as the lungs, heart, and blood (Semenza, 2004). Studies at the cellular level indicate that HIF directly or indirectly regulates several hundred genes that serve a great variety of functional pathways including angiogenesis, cell growth, apoptosis, energy metabolism and vasomotor regulation (Semenza, 2004). Recent studies in humans have also demonstrated a major role in systemic responses to hypoxia that extends to each of the principal organ systems upon which cellular oxygen delivery ultimately depends. This article reviews our current understanding of the importance of HIF in human systems-level physiology, and is modelled around the classic physiological response to high-altitude hypoxia. Readers are referred to other reviews for detailed analyses of the interaction between HIF and cancer biology (Patiar & Harris, 2006), angiogenesis (Hirota & Semenza, 2006) and therapeutics (Hewitson & Schofield, 2004; Semenza, 2006).

## Integrative human hypoxia physiology: the response to high altitude

Lowland residents travelling to high altitude experience profound physiological changes as a result of environmental hypoxia. This acclimatisation process conveniently demonstrates the systemic effects of isolated hypoxia, which are less pleiotropic than might be expected from the great functional diversity present within the HIF-regulated transcriptome. Haematological, respiratory and cardiovascular responses predominate and are described in this section.

### Erythropoietic response to hypoxia

**Fig 1:**
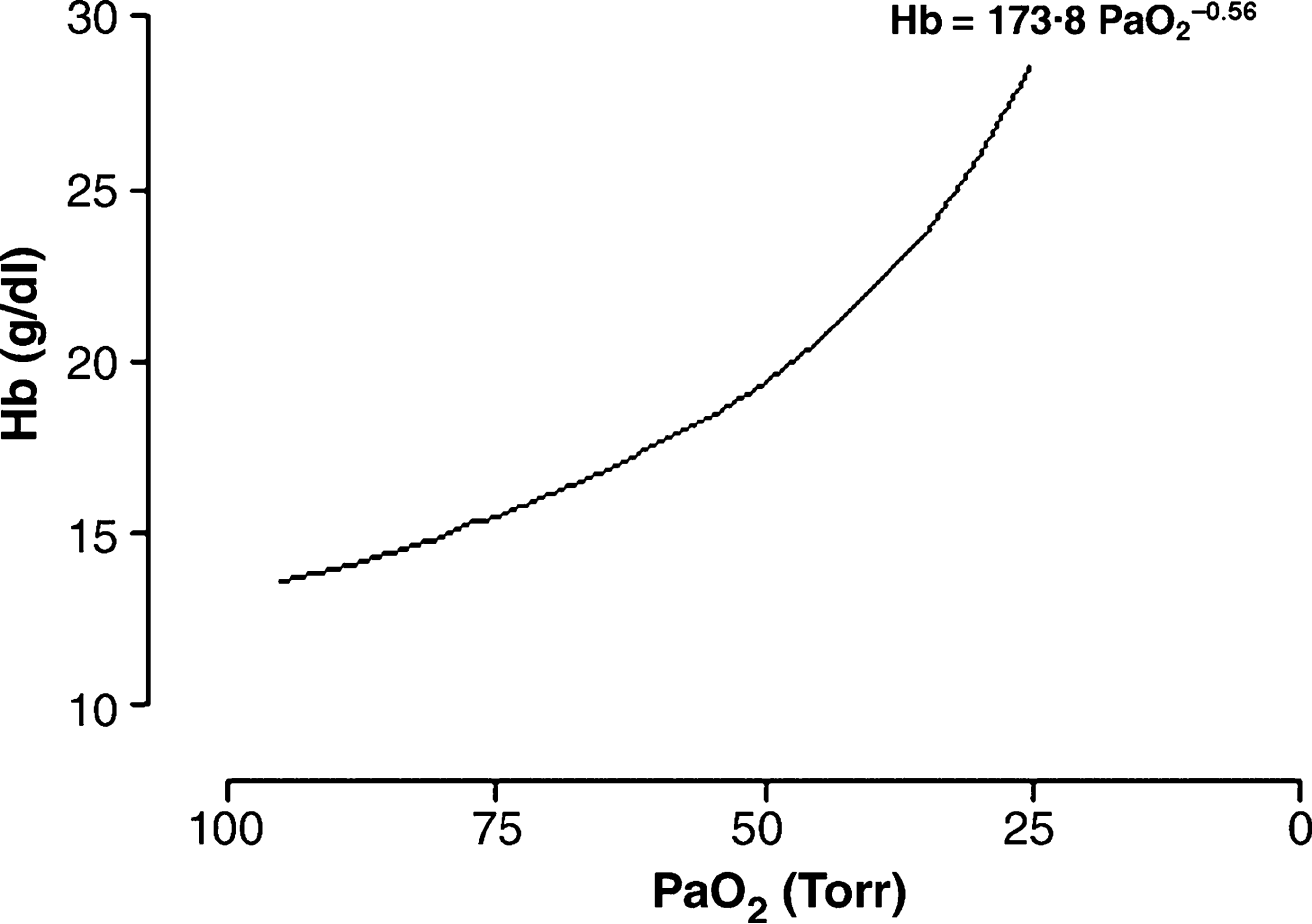
Empirically-derived relationship between haemoglobin concentration (Hb) and arterial partial pressure of oxygen (Pao_2_) in Andean men. From Villafuerte *et al* (2004) (used with permission). The average Hb of young high-altitude natives is represented by an empirical equation that expresses Hb as a function of Pao_2_. This equation was derived using data collated from several studies, which investigated approximately 200 healthy men aged 18–45 years. Measurements were largely made in members of the native Quechua population at altitudes ranging from sea level to 4860 m, and Pao_2_ was calculated from arterial haemoglobin oxygen saturation. Pao_2_ is primarily determined by the altitude of residence, and for a given altitude, a Hb of more than two standard deviations above average is considered excessive (Villafuerte *et al*, 2004).

Though slower in onset than its cardiopulmonary counterparts, the development of polycythaemia has been the most widely known feature of acclimatisation since Viault first reported a proliferative effect of high altitude on his own red cell count (Viault, 1890). After years of subsequent conjecture, the existence of a humoral erythropoietic factor was eventually proven in rabbits by transfusing normal animals with plasma from severely anaemic donors (Erslev, 1953), although human Epo was not isolated at sufficient purity to obtain a partial protein sequence until 1977. Hypoxia generates a detectable increase in serum Epo within 90 min (Eckardt *et al*, 1989). Following ascent to high altitude, Epo levels peak within 2 d and thereafter decline towards sea-level values over a period of 1–2 weeks (Abbrecht & Littell, 1972; Milledge & Cotes, 1985; Richalet *et al*, 1994). This decline occurs despite ongoing hypoxia and notably precedes most of the expansion of red cell mass. Haemoglobin (Hb) and haematocrit (Hct) are nevertheless elevated within days of ascent (Richalet *et al*, 1994) and red cell mass has been shown to continue rising for up to eight months and, for an altitude of 4500 m, approach a total increase of 50% (Reynafarje *et al*, 1959). The polycythaemic response is therefore largely developed and maintained in the presence of relatively normal levels of Epo, as is the case when secondary polycythaemia results from hypoxic lung disease (Wedzicha *et al*, 1985). The maximum expansion in red cell mass varies with the severity of hypoxia – at extreme altitudes, Hb > 210 g/l and Hct > 60% have been reported in acclimatising individuals (Winslow *et al*, 1984). Values are naturally higher in high altitude natives, and for Andean males the normal Hb has been related to the arterial partial pressure of oxygen through an empirically-derived function, as depicted in Fig 1. Because of this relationship, the magnitude of erythrocytosis that is considered excessive and defines chronic mountain sickness varies with altitude.

### Ventilatory acclimatisation to hypoxia

Pulmonary ventilation is closely coupled to energy metabolism in the body, simultaneously providing an essential substrate (oxygen) and removing a major by-product (carbon dioxide), and ventilation is duly influenced by changes in the arterial partial pressures of both oxygen (Po_2_) and carbon dioxide (Pco_2_). Experimental investigations into the respiratory effects of high altitude hypoxia date back to Angelo Mosso's pioneering experiments in the Italian Alps in the 1890's (West, 1998). In 1911 the landmark Oxford-Yale expedition to Pike's Peak, Colorado (altitude 4300 m), confirmed the earlier observation that alveolar Pco_2_ declines at altitude and demonstrated an increase in alveolar Po_2_ as a result of hyperventilation (Douglas *et al*, 1913). It is now well known that acute hypoxia stimulates an immediate increase in minute ventilation that peaks within minutes and then declines towards prehypoxic levels over the following minutes-to-hours. When hypoxia is sustained, as at high altitude, ventilation begins to rise again after an hour or two, soon surpassing acute hypoxic levels and continuing to rise for several days. This progressive increase in ventilation is known as ventilatory acclimatisation to hypoxia, and the resultant elevation in arterial Po_2_ mitigates the impact of environmental hypoxia. The characteristic features of ventilatory acclimatisation include a concomitant fall in Pco_2_, and an increase in hypoxic ventilatory sensitivity that heightens the ventilatory response to any further acute hypoxic stimulus (Douglas *et al*, 1913; Rahn & Otis, 1949; Sato *et al*, 1992).

### Hypoxia-induced changes in cardiac output

Acute hypoxia stimulates a reflex increase in cardiac output. Pike's Peak also provided the setting for the first high altitude measurements of cardiac output, where the acetylene gas uptake technique was used to demonstrate a progressive increase in cardiac output over several days (Grollman, 1930). This increase is associated with an elevation in heart rate rather than stroke volume (Vogel & Harris, 1967). Cardiac output gradually normalises as individuals acclimatise, although heart rate may remain high accompanied by a lower stroke volume.

### Pulmonary vascular acclimatisation to hypoxia

In 1947, right heart catheterisation of volunteers breathing 10% oxygen provided the first evidence that, in contrast with other vascular beds, hypoxia constricts rather than dilates the human pulmonary vasculature (Motley *et al*, 1947). Hypoxia acutely elevates pulmonary arterial pressure, which reaches a plateau within 5 min (Talbot *et al*, 2005). Pulmonary arterial pressure begins to increase again after approximately 45 min (Talbot *et al*, 2005) and continues to rise for at least another 2 h (Dorrington *et al*, 1997). Pulmonary vascular acclimatisation is characterised by the resultant elevation in pulmonary arterial pressure, and by an accompanying increase in the sensitivity of the pulmonary vascular response to any additional acute hypoxic stimulus (Dorrington *et al*, 1997).

Although this pulmonary hypertensive response is a prominent consequence of ascent to high altitude, unlike other features of acclimatisation it is generally troublesome rather than beneficial. The underlying phenomenon of hypoxic pulmonary vasoconstriction is of great importance during foetal life. In utero, pulmonary perfusion needs only meet the metabolic demands of the lung tissue itself, and vasoconstriction in the relatively hypoxic unventilated lung is instrumental in shunting oxygenated blood to the rest of the body via the foetal circulation. Following delivery and aeration of the lungs, rapid reversal of pulmonary vasoconstriction facilitates transition to the adult circulation, in which the systemic and pulmonary vasculature are anatomically ‘in series’ and thus virtually the entire cardiac output can participate in gas exchange. In adults, regional hypoxic pulmonary vasoconstriction can be beneficial in matching perfusion to ventilation throughout the lung (eg in single lung anaesthesia), although under normal conditions its contribution to maintaining Po_2_ over the physiological range remains uncertain (Dorrington & Talbot, 2004). Global hypoxic pulmonary vasoconstriction involving the entire lung is commonly undesirable as it eventually leads to pulmonary vascular remodelling and pulmonary hypertension, which frequently complicates hypoxic lung disease and worsens survival of patients with conditions such as chronic obstructive pulmonary disease (Barbera *et al*, 2003). Hypoxia-induced pulmonary hypertension is also a major cause of morbidity in high altitude residents, where it manifests in chronic and subacute mountain sickness, while in non-residents it can acutely precipitate high altitude pulmonary oedema, the most common cause of death related to high altitude (Hackett & Roach, 2001; Penaloza & Arias-Stella, 2007).

## HIF transcriptional activation system

Over the past 15 years the role of HIF in cellular oxygen signalling has been extensively investigated. The first member of the HIF family of transcription factors to be discovered, HIF-1, was originally identified as a hypoxia-inducible nuclear factor that bound to the hypoxia-response element enhancer region of the Epo gene (Semenza & Wang, 1992). In accordance with the effects of hypoxia on serum Epo, the DNA-binding activity of HIF-1 was found to be tightly regulated by cellular oxygen tension. Subsequent purification revealed that HIF-1 was in fact a heterodimer consisting of HIF-1α and HIF-1β subunits that are both members of the basic helix-loop-helix PAS protein family (Wang *et al*, 1995). Studies in cultured cells found that cobalt chloride had a hypoxia-mimetic effect on HIF-1 and expression of Epo RNA (Wang & Semenza, 1993a), consistent with previous observations that cobalt causes polycythaemia in humans. Desferrioxamine was also found to induce HIF-1 activity and *EPO* RNA expression with kinetics similar to that of induction by hypoxia and cobalt chloride (Wang & Semenza, 1993b). Although initially studied extensively in the context of Epo regulation, HIF-1 and its recognition sequence were soon shown to be common components of a general mammalian cellular response to hypoxia. Early studies demonstrated oxygen-dependent DNA binding of HIF and transcriptional activation of a hypoxia response element in a variety of cell lines (Maxwell *et al*, 1993; Wang & Semenza, 1993a) and for a variety of genes (Firth *et al*, 1994). Further experiments determined that HIF-1β is constitutively expressed, is present in excess, is a component of other transcription factors and is unaffected by hypoxia, whereas HIF-1α is unique to HIF-1 and is the primary determinant of *HIF-1* DNA binding and transcriptional activity (Huang *et al*, 1996; Jiang *et al*, 1996; Semenza *et al*, 1996). As the list of HIF-regulated genes continued to grow steadily, more detailed analyses determined that, under euoxic conditions, HIF-1α is synthesised continuously but is rapidly degraded by the ubiquitin-proteasome system via a process that requires the presence of two sequences in HIF-1α termed oxygen-dependent degradation domains (Huang *et al*, 1996, 1998; Pugh *et al*, 1997; Salceda & Caro, 1997). In addition to the HIF-1α protein, two closely related proteins that also dimerise with HIF-1β were identified termed HIF-2α (Tian *et al*, 1997) and HIF-3α (Gu *et al*, 1998). HIF-2α has a more restricted expression profile than HIF-1α but has many similar functional characteristics (Wiesener *et al*, 1998), while the regulation and functions of HIF-3α are less well understood.

By this stage it was known that hypoxic stabilisation of HIF-α subunits leads to their accumulation, but the oxygen sensor responsible for regulating this process remained elusive. A major advance followed from the recognition that HIF-target genes are upregulated in tumours of the von Hippel-Lindau inherited cancer syndrome, in which the von Hippel-Lindau tumour suppressor protein (VHL) is mutated. Subsequent research showed that VHL binds to the HIF-α subunit and targets it for ubiquitin-mediated proteasomal degradation by acting as the recognition component of a multiprotein ubiquitin ligase complex (Maxwell *et al*, 1999; Cockman *et al*, 2000; Ohh *et al*, 2000). In 2001 the fundamental oxygen-sensitive step underlying the degradation of HIF-α was defined as oxygen-dependent hydroxylation of specific proline residues in HIF-α, a modification that directs the binding of VHL (Ivan *et al*, 2001; Jaakkola *et al*, 2001; Yu *et al*, 2001). Three isoenzymes, termed prolyl hydroxylase-domain (PHD) proteins, were subsequently identified that catalyse this prolyl hydroxylation of HIF-α (Epstein *et al*, 2001). The three PHDs (PHD1, PHD2 and PHD3) are members of the 2-oxoglutarate- and iron-dependent dioxygenase family of enzymes and their activity is dependent upon oxygen as a co-substrate together with iron, ascorbate and 2-oxoglutarate as obligate cofactors.

Thus, under euoxic conditions HIF-α is continuously expressed and rapidly degraded whereas under hypoxic conditions, prolyl hydroxylation and proteasomal degradation are slowed, resulting in stabilisation and accumulation of HIF-α. The HIF-α subunit is then translocated to the nucleus where it dimerises with HIF-β, binds to the hypoxia response elements of HIF-target genes and activates their transcription.

**Fig 2:**
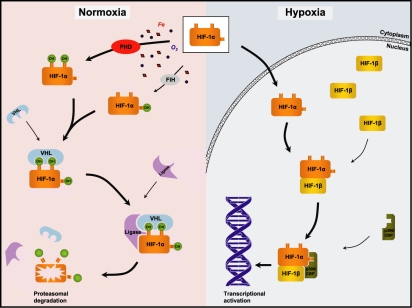
Schematic representation of the PHD-VHL-HIF axis. The hypoxia-inducible factor (HIF)-α subunit is synthesised continuously but is rapidly destroyed in the presence of oxygen and iron. Oxygen- and iron-dependent prolyl hydroxylase domain (PHD) enzymes hydroxylate specific proline residues in HIF-α, increasing its affinity for the von Hippel-Lindau tumour suppressor protein (VHL). The binding of VHL to hydroxylated HIF-α then targets HIF-α for destruction by a multiprotein ubiquitin ligase (denoted ‘ligase’) that mediates proteasomal degradation of HIF-α subunits. Another oxygen- and iron-dependent enzyme, factor inhibiting HIF (FIH), hydroxylates an asparagine residue in HIF-α, reducing its ability to activate transcription by inhibiting binding of the transcriptional coactivator complex p300/CBP. Under hypoxic conditions, the hydroxylation of HIF-α by PHDs and FIH is inhibited and proteasomal degradation is thus slowed. HIF-α rapidly accumulates and dimerises with HIF-β, which is expressed constitutively and is present in excess. The p300/CBP coactivator is recruited, and the DNA binding complex subsequently up-regulates hypoxia-responsive genes. OH denotes hydroxyl group.

Hypoxia-inducible factor-α undergoes a further post-translational modification that affects its transcriptional activity rather than its stability. Hydroxylation of an asparagine residue within the C-terminal transactivation domain of HIF-α reduces its transcriptional activity by inhibiting binding of the transcriptional coactivator complex p300/CBP (Hewitson *et al*, 2002; Lando *et al*, 2002). This asparaginyl hydroxylase, termed factor inhibiting HIF (FIH), also belongs to the 2-oxoglutarate- and iron-dependent dioxygenase superfamily and is similarly oxygen-dependent. Normal mammalian cell and tissue oxygen tensions fall within the sensitive range of PHD enzyme kinetics, such that small changes in oxygen tension cause significant changes in enzymatic catalysis (Epstein *et al*, 2001; Hirsila *et al*, 2003). FIH appears to have a ‘fine-tuning’ role secondary to that of the PHDs, exerting an additional level of negative control on HIF-α that has escaped degradation (Stolze *et al*, 2004). Although other signalling pathways interact with HIF and affect the ultimate transcriptional response to hypoxia, the PHD-VHL-HIF axis is now firmly established as the central regulator of cellular oxygen homeostasis and is illustrated schematically in Fig 2.

## Initial evidence implicating HIF in systemic physiology

The discovery of HIF, its ubiquity and the functional diversity of its many target genes has inspired researchers to explore its wider influence in systemic physiology. Early insights were gained from experiments using genetically engineered mammalian models and from studies manipulating HIF pharmacologically in humans. In mice exposed to sustained hypoxia, heterozygous deficiency for functional HIF genes delays the development of polycythaemia (Yu *et al*, 1999) and prevents normal ventilatory acclimatisation to both chronic hypoxia (Kline *et al*, 2002) and intermittent hypoxia (Peng *et al*, 2006). The development of hypoxia-induced pulmonary hypertension and vascular remodelling are also markedly impaired in such mice (Yu *et al*, 1999; Brusselmans *et al*, 2003), as are electrophysiological responses to hypoxia in the pulmonary arterial myocytes responsible for these processes (Shimoda *et al*, 2001).

Through intravenous infusions of desferrioxamine (DFO), studies in humans sought to modify putative HIF-dependent physiological processes by exploiting the hypoxia-mimetic effect that iron chelation has on HIF hydroxylation. In a study of 16 subjects, an 8-h infusion of DFO increased serum Epo in a dose-dependent manner but did not measurably affect ventilatory sensitivity to acute hypoxia within 24 h of commencing the infusion (Ren *et al*, 2000). However, research in rats suggests that ventilatory effects may not be detectable until 2–3 d after administration of DFO (Nguyen *et al*, 2007). In a further study, pulmonary vascular tone was assessed echocardiographically in 20 subjects either during an infusion of DFO or during exposure to hypoxia, and DFO was seen to induce a mild elevation of pulmonary arterial pressure over a similar time course to that associated with hypoxia (Balanos *et al*, 2002).

Together these studies in animals and humans provided indirect supportive evidence that HIF is important in human cardiopulmonary physiology. In particular, these studies introduced the possibility that HIF-regulated induction of gene expression might mediate physiological responses to sustained hypoxia, and that basal HIF activity might calibrate the set point and sensitivity of systemic responses to an acute hypoxic stimulus.

## HIF and systemic human physiology

### HIF and erythropoiesis

Much of what is known about the importance of HIF in systemic human physiology has been learnt from phenotyping haematology patients diagnosed with rare monogeneic disease. The most extensively studied of these is Chuvash polycythaemia, a rare autosomal recessive disorder that was originally described in the Chuvash population of Russia (Polyakova, 1974). Affected individuals are homozygous for a specific *598C>T* mutation in the von Hippel-Lindau tumor suppressor gene (*VHL*) that results in an amino acid change of arginine to tryptophan (Ang *et al*, 2002). This diminishes the binding affinity of VHL for hydroxylated HIF-α, thereby subtly inhibiting HIF-α degradation and pathologically activating HIF target genes (Ang *et al*, 2002). The mutation is endemic to Chuvashia and also to the Italian island of Ischia (Sergeyeva *et al*, 1997; Perrotta *et al*, 2006), and has been identified as a cause of congenital polycythaemia in individuals from many other ethnic backgrounds (Percy, 2007). With the exception of one individual, in all cases the *VHL 598C>T* mutation has been associated with the same haplotype, indicating a common founder (Percy, 2007). Patients usually present with symptoms of polycythaemia in early adulthood or are diagnosed on family screening, and are typically asymptomatic following treatment with therapeutic venesection. Chuvash polycythaemia is associated with early mortality, due partly to thrombotic complications, and carries a predisposition towards benign vertebral body haemangiomas, varicose veins and low systemic blood pressure (Gordeuk *et al*, 2004). The condition has features of both primary and secondary polycythaemia, as serum Epo is inappropriately raised yet erythroid progenitors are also hypersensitive to Epo (Ang *et al*, 2002). Over-expression of many HIF-regulated gene products has been documented, including Epo, vascular endothelial growth factor, glucose uptake transporter-1, transferrin, transferrin receptor, plasminogen activator inhibitor-1, aldolase C and endothelin-1 (Ang *et al*, 2002; Gordeuk *et al*, 2004; Bushuev *et al*, 2006; Smith *et al*, 2006).

In addition to the Chuvash mutation, several other *VHL* mutations have also been associated with congenital polycythaemias (Percy, 2007), and it has been stated that *VHL* mutations represent the most frequent cause of congenital polycythaemia (Gordeuk *et al*, 2005). These polycythaemia-associated mutations do not overlap with the many *VHL* mutations that are known to cause the classic VHL cancer syndrome, which is an entirely distinct disease and is only rarely complicated by polycythaemia (Lonser *et al*, 2003). Another form of familial polycythaemia caused by dysregulation of HIF signalling has recently been identified (Percy *et al*, 2006). In three related individuals, a novel mutation in *EGLN1* (which encodes PHD2) was associated with polycythaemia and inappropriately normal levels of Epo. Several further *EGLN1* mutations associated with polycythaemia have since been described in unrelated individuals (Al-Sheikh *et al*, 2007; Percy *et al*, 2007). Heterozygosity for these mutations appears to reduce the functional activity of PHD2 (Percy *et al*, 2006), which is the principal PHD isoenzyme regulating the low steady-state level of HIF-α under euoxic conditions (Berra *et al*, 2003).

These rare manifestations of genetic disease together confirm the importance of the PHD-VHL-HIF axis in controlling Epo expression and thus red cell mass in humans. However, the response to Epo stimulation is dependent upon maintenance of sufficient marrow availability of rate-limiting substrates. Though Hb is increased within a week of ascent to high altitude, some authors have observed a transient fall in Hb over the following 1–2 weeks, when serum ferritin is sharply lowered (Richalet *et al*, 1994). These authors suggest that this fall in Hb results from intercurrent iron deficiency and consequent marrow resistance to Epo, and that the polycythaemic response resumes upon restoration of adequate iron availability (Richalet *et al*, 1994). It is therefore not surprising that hypoxia has long been known to increase intestinal iron absorption in humans adapting to high altitude (Reynafaire & Ramos, 1961) and that HIF promotes iron transport by up-regulating transferrin (Rolfs *et al*, 1997) and the transferrin receptor (Lok & Ponka, 1999; Tacchini *et al*, 1999). However, the coordinated mechanism through which hypoxia-induced erythropoietic iron demands are met has only recently been elucidated.

Though better known for inducing the expression of its transcriptional targets, HIF also represses a small number of genes (eg Chen *et al*, 2005). Recent experiments in genetically engineered mice have shown that HIF negatively regulates hepcidin, the key hormonal regulator of iron homeostasis (Peyssonnaux *et al*, 2007). Hepcidin is synthesised in the liver and inhibits active transmembrane export of iron by blocking ferroportin, the sole iron-transporter responsible for iron efflux from enterocytes and macrophages (Nemeth *et al*, 2004). Down-regulation of hepcidin liberates ferroportin, thereby increasing intestinal iron absorption and mobilising recycled iron from macrophages (Ganz, 2006). Through the stabilisation of HIF-α, hypoxia and iron-deficiency both suppress hepcidin and thereby increase iron availability, at least in mice (Peyssonnaux *et al*, 2007). Similar involvement of HIF in humans seems likely, as hepcidin is likewise critical to human iron homeostasis – indeed, hepcidin deficiency underlies most forms of the iron-overload disorder hereditary haemochromatosis (Ganz, 2006). Thus, it appears that HIF simultaneously stimulates the polycythaemic response to hypoxia and boosts the availability of iron so as to meet the resultant erythropoietic demands.

Iron and HIF are further coupled through the action of iron regulatory proteins IRP1 and IRP2. IRPs post-transcriptionally regulate the expression of several proteins of iron metabolism by binding to their mRNA iron-responsive elements (IREs). When iron is scarce, IRP-IRE binding promotes cellular iron uptake and prevents iron sequestration by stabilising transferrin receptor mRNA and inhibiting the translation of ferritin mRNA. In contrast, in iron-replete cells IRP1 is biochemically inactivated and IRP2 undergoes iron-dependent proteasomal degradation, resulting in reduced IRE-binding activity and opposite homeostatic effects. It is interesting to note that it has been proposed that the IRP2 degradation pathway involves an unidentified 2-oxoglutarate-dependent dioxygenase from the same family as the HIF hydroxylases (Wang *et al*, 2004), although VHL is not a necessary component of this pathway (Wang & Pantopoulos, 2005). Furthermore, it has recently been reported that HIF-2α is post-translationally regulated by IRP-IRE binding (Sanchez *et al*, 2007). Since HIF-2α appears to be the most important HIF isoform in regulating Epo (Rankin *et al*, 2007), this introduces a negative-feedback control mechanism that may operate during iron-deficiency to limit Epo stimulation of erythropoiesis until iron availability is sufficient to allow effective red blood cell production.

### HIF and ventilation

Ventilatory responses to acute hypoxia are mediated by the glomus cells of the carotid body. Though the precise oxygen-sensing mechanism has yet to be determined, it is generally accepted that hypoxic inhibition of potassium channels leads to membrane depolarisation, calcium influx, release of neurotransmitters and stimulation of sensory nerves that activate the respiratory centre of the brain (Weir *et al*, 2005). The control of ventilation is not well understood, and the underlying biology of ventilatory acclimatisation to hypoxia remains a subject of debate. However, the hypothesis now gaining most credence contends that ventilatory acclimatisation is driven by hypoxia-induced changes in carotid body function that involve oxygen-regulated alterations in gene expression (Robbins, 2007). A recent set of experiments conducted in patients with Chuvash polycythaemia strongly implicate HIF in the control of ventilation (Smith *et al*, 2006).

Three venesected subjects with Chuvash polycythaemia were compared with matched ‘normal’ healthy control subjects, and with venesected ‘polycythaemic’ control subjects who had each been diagnosed with forms of polycythaemia unrelated to oxygen-sensing. While breathing air, average arterial Pco_2_ was significantly lower in the Chuvash polycythaemia patients, indicating an altered set point for respiratory control in the direction of an elevation in pulmonary ventilation. Cardiopulmonary responses to isocapnic hypoxia were assessed using the technique of dynamic end-tidal forcing while subjects breathed on a mouthpiece system. The ventilatory response stimulated by acute hypoxia was several-fold greater in the Chuvash polycythaemia patients than in controls for both mild and moderate hypoxic stimuli. Thus, patients with Chuvash polycythaemia had both a reduced Pco_2_ set point for the respiratory controller and an abnormally high acute hypoxic ventilatory sensitivity, which together are characteristic of acclimatised individuals. These results strongly support the notion that HIF is involved in human ventilatory acclimatisation to hypoxia, and establish the importance of HIF in respiratory control in hypoxia-naïve individuals. Such involvement of HIF is consistent with the nature of some of the genes HIF is known to activate, most notably tyrosine hydroxylase, which regulates the biosynthesis of catecholamine neurotransmitters in the rat carotid body and brainstem respiratory centre (Nguyen *et al*, 2007).

### HIF and the pulmonary vasculature

The mechanism underlying hypoxic pulmonary vasoconstriction is not completely understood, although it is known to arise from the inherent properties of pulmonary arteriolar smooth muscle cells and is modulated by a variety of endothelial-derived vasoactive mediators. The initial sequence is somewhat analogous to that of the carotid body glomus cells – in contrast with smooth muscle cells from other vascular beds, hypoxic inhibition of outward potassium current causes depolarisation of the pulmonary arterial myocyte membrane, resulting in calcium entry and subsequent contraction (Weir *et al*, 2005). However, the oxygen sensor or sensors that activate this sequence, and the ion channels and signal transduction pathways involved, remain a source of much scientific controversy (Aaronson *et al*, 2006).

In the same study of patients with Chuvash polycythaemia, a standard echocardiographic technique was used to determine pulmonary vascular tone at baseline and during exposure to hypoxia (Smith *et al*, 2006). Chuvash polycythaemia patients were found to have a degree of pulmonary hypertension, with basal systolic pulmonary arterial pressures exceeding 30 mmHg. This result is consistent with other reports (Bushuev *et al*, 2006) and most likely reflects HIF-regulated elevation in pulmonary arterial pressure (Smith *et al*, 2006). The patients also displayed exquisite pulmonary vascular sensitivity to hypoxia, with hypoxic responses 5- to 10-fold greater than those of controls. Indeed, a moderate hypoxic stimulus (end-tidal Po_2_ of 50 mmHg) provoked peak systolic pulmonary arterial pressures exceeding 70 mmHg in the patients, compared with less than 30 mmHg in the control group. Several HIF-activated genes are important in vasomotor regulation and may be involved in the pulmonary vascular response to hypoxia (Schofield & Ratcliffe, 2004; Smith *et al*, 2006). The best studied of these is the vasoconstrictor endothelin-1 (*EDN1*), which is elevated in patients with Chuvash polycythaemia (Bushuev *et al*, 2006) and in the lungs of patients with pulmonary hypertension (Giaid *et al*, 1993).

## Variation within the PHD-VHL-HIF axis

The profound physiological disturbances associated with the Chuvash mutation raise the question of whether common genetic variation within the PHD-VHL-HIF axis might also have phenotypic effects on physiological function. Such effects might explain some of the considerable inter-individual variation seen in Epo secretive (Richalet *et al*, 1994), ventilatory (Hirshman *et al*, 1975) and pulmonary vascular responses (Groves *et al*, 1993) to hypoxia. It is therefore interesting that specific polymorphic variants of *HIF1A* have been proposed to be associated with maximal oxygen consumption during exercise (Prior *et al*, 2003), and that certain *HIF1A* genotypes have been reported to occur more frequently in the Sherpa population of Tibet than in lowlanders (Liu *et al*, 2007). However, a study of Andean high-altitude natives found no associations between severe polycythaemia and polymorphisms of several genes involved in hypoxia sensing, including the major components of the PHD-VHL-HIF axis (Mejia *et al*, 2005).

Another potential source of variation in HIF activity is iron status. Iron is an obligate cofactor in the hydroxylation reaction through which HIF is primarily regulated, and in cultured cells HIF degradation is both inhibited by iron chelation with DFO and potentiated by supraphysiological iron supplementation (Wang & Semenza, 1993b; Knowles *et al*, 2003). Iron availability might similarly affect HIF degradation in humans and thereby influence HIF-dependent processes. This appears to have been the case in the DFO infusion experiments described previously, and it has been suggested that in Chuvash polycythaemia, venesection-induced iron deficiency could phenocopy the physiological manifestations of VHL loss of function to some extent (Smith *et al*, 2006). A further interesting possibility is suggested by the observation that iron supplementation may increase the incidence of pre-eclampsia – a disease postulated to be caused by inadequate placental development (Ziaei *et al*, 2007). Iron-induced down-regulation of HIF might in theory explain this otherwise puzzling association (Smith & Robbins, 2007).

## Conclusion

Hypoxic regulation of erythropoiesis provided the paradigm through which HIF was discovered and initially investigated. Convergent discoveries in the biochemistry of oxygen sensing and in cardiopulmonary physiology have since established that the PHD-VHL-HIF axis is important in regulating both intracellular and systems-level human biology. The emerging role of HIF in systemic physiology, described here in terms of the response to high altitude, in fact translates to any clinical scenario in which hypoxia is a feature. Functional differences arising from common genetic variation or the availability of cofactors, such as iron, may therefore influence the clinical response to any such physiological or pathological disturbance.
